# Disparities in brain health comorbidity management in intracerebral hemorrhage

**DOI:** 10.3389/fneur.2023.1194810

**Published:** 2023-06-08

**Authors:** Ernst Mayerhofer, Natalie O. Zaba, Livia Parodi, Alena S. Ganbold, Alessandro Biffi, Jonathan Rosand, Nirupama Yechoor, Christopher D. Anderson

**Affiliations:** ^1^Center for Genomic Medicine, Massachusetts General Hospital, Boston, MA, United States; ^2^Henry and Allison McCance Center for Brain Health, Massachusetts General Hospital, Boston, MA, United States; ^3^Program in Medical and Population Genetics, Broad Institute of Harvard and MIT, Massachusetts Institute of Technology, Cambridge, MA, United States; ^4^Department of Neurology, Brigham and Women’s Hospital, Boston, MA, United States

**Keywords:** intracerebral hemorrhage, social determinants of health, hyperlipidemia, diabetes, obstructive sleep apnea, hearing impairment

## Abstract

**Background:**

Intracerebral hemorrhage (ICH) disproportionally affects underserved populations, and coincides with risk factors for cardiovascular events and cognitive decline after ICH. We investigated associations between social determinants of health and management of blood pressure (BP), hyperlipidemia, diabetes, obstructive sleep apnea (OSA), and hearing impairment before and after ICH hospitalization.

**Methods:**

Survivors of the Massachusetts General Hospital longitudinal ICH study between 2016 and 2019 who received healthcare at least 6 months after ICH were analyzed. Measurements of BP, LDL and HbA1c and their management in the year surrounding ICH and referrals for sleep studies and audiology up to 6 months after ICH were gathered from electronic health records. The US-wide area deprivation index (ADI) was used as proxy for social determinants of health.

**Results:**

The study included 234 patients (mean 71 years, 42% female). BP measurements were performed in 109 (47%) before ICH, LDL measurements were performed in 165 (71%), and HbA1c measurements in 154 (66%) patients before or after ICH. 27/59 (46%) with off-target LDL and 3/12 (25%) with off-target HbA1c were managed appropriately. Of those without history of OSA or hearing impairment before ICH, 47/207 (23%) were referred for sleep studies and 16/212 (8%) to audiology. Higher ADI was associated with lower odds of BP, LDL, and HbA1c measurement prior to ICH [OR 0.94 (0.90–0.99), 0.96 (0.93–0.99), and 0.96 (0.93–0.99), respectively, per decile] but not with management during or after hospitalization.

**Conclusion:**

Social determinants of health are associated with pre-ICH management of cerebrovascular risk factors. More than 25% of patients were not assessed for hyperlipidemia and diabetes in the year surrounding ICH hospitalization, and less than half of those with off-target values received treatment intensification. Few patients were evaluated for OSA and hearing impairment, both common among ICH survivors. Future trials should evaluate whether using the ICH hospitalization to systematically address co-morbidities can improve long-term outcomes.

## Introduction

Intracerebral hemorrhage (ICH) and its sequelae have a disproportionally high impact on minority populations in the United States (1). Disparities in access to health care and socioeconomic factors limit the ability of marginalized populations to receive adequate care before and after ICH (2–5). The incidence and recurrence of ICH is higher in people of Black and Hispanic racial and ethnic backgrounds (3, 4), who are also at elevated risk of cognitive impairment, at least partially due to their higher burden of underlying cerebrovascular risk factors (6, 7). Blood pressure (BP) management, crucial for ICH prevention (8), is less effective in neighborhoods with low income, pointing to possible inequities in postdischarge care (2, 5).

Because treatment options for ICH remain limited, focus lies in prevention and rehabilitation (9). BP control is the most important risk factor for ICH incidence and recurrence, but after ICH, survivors are also at high risk of ischemic stroke (10), myocardial infarction (10), and incident dementia (11). However, targeted prevention efforts toward these common comorbidities are currently not prioritized (9). Obstructive sleep apnea (OSA) is present in up to 80% of ICH patients, raises BP, and is a significant risk factor for ICH and cardiovascular disease (1, 12). Hearing impairment is present in approximately 70% of individuals with stroke (13), and is associated with lower likelihood of good functional and cognitive outcomes after ICH (14).

Current guidelines do not recommend goal-directed work-up for risk factors and comorbidities that are not directly related to ICH (9). Although evidence from clinical trials is missing, diagnosis and treatment of common cardiovascular and brain health risk factors may contribute to improved overall outcomes after ICH. In this study, we sought to understand current clinical practices for ICH patients and whether social determinants of health are associated with providers’ management rates for selected risk factors and comorbidities that are associated with adverse outcomes in ICH survivors. In addition to blood pressure, we chose hyperlipidemia and diabetes as cerebrovascular risk factors [screened by low-density lipoprotein (LDL) and glycated hemoglobin (HbA1c), respectively], two conditions that have well-established management strategies to improve vascular outcomes even if not directly related to the ICH that resulted in the hospitalization (15, 16). OSA and hearing impairment were additionally selected for this study as they are common comorbidities among ICH survivors (1, 14) with widely available diagnostics and treatment that can impact recurrence, recovery, and cognition after stroke (14, 17, 18). Additionally, these conditions often remain underdiagnosed and inadequately managed, thereby representing important areas for potential intervention to improve patient outcomes following an ICH event (19–21). We assessed measurement and management rates of these conditions before, during, and after ICH hospitalization in a population admitted to a tertiary care medical center and assessed their associations with measures of social disadvantage.

## Methods

### Study cohort

ICH admission data was gathered from the Massachusetts General Hospital (MGH) ICH study, a prospective and consecutive study including all patients presenting with ICH at MGH between 1991 and 2019 (8). To exclude potential bias from different electronic health record data sources, data was restricted to individuals that were admitted after October 2016, 6 months after Epic (Epic Systems, Verona, WI) was introduced as electronic health record (EHR) software at MGH and affiliated providers. Patients aged 18 years or older with acute primary ICH confirmed by CT scan admitted within 24 h after symptom onset were included. Patients with ICH resulting from trauma, conversion of ischemic infarct, vascular malformation or aneurysm, or brain tumor were excluded. The study was approved by the MGH institutional review board (#2021P001597) and informed consent was obtained from all participants or their authorized surrogates.

We investigated patient’s health care in the 6 months before hospitalization for ICH, during hospitalization (14 days following ICH), and in the 6 months after hospitalization. We included only individuals who survived at least 6 months after ICH event to allow sufficient time for post-ICH testing. To capture only individuals whose health care data is reflected in the electronic health record (EHR), those without a recorded encounter 6 months or later after ICH were excluded. Measurements of BP, LDL, and HbA1c, as well as referrals and appointments for audiology, sleep medicine, and home or facility-based sleep studies within the 12 months surrounding ICH were gathered from the EHR. To identify patients with prior diagnoses of OSA or hearing impairment, diagnoses, problem lists, and clinical notes were searched for discrete diagnoses or descriptive text (Supplementary Methods).

### Evaluation of management

BP prescriptions were ascertained by gathering all medication prescriptions of the patient cohort in the 12 months surrounding ICH, and assigning every BP medication into one of five different classes: calcium channel blockers, beta blockers, ace inhibitors/angiontensin receptor antagonists, diuretics, and others (hydralazine, alpha blockers). For each patient, we assigned the number of antihypertensive drug classes prescribed in the 6 months before and in the 6 months after ICH (excluding the acute inpatient treatment 14 days after ICH), as well as the number of antihypertensive drugs initiations in the 6 months before ICH. Intensification of antihypertensive medication was not analyzed due to unavailable dosage data in the EHR.

We defined a target LDL of <70 mg/dL for patients with a documented history of coronary artery disease (CAD), ischemic stroke, TIA, and diabetes at the time of measurement, otherwise <190 mg/dL. We defined treatment as appropriate or inappropriate depending on medication prescriptions before and after LDL measurement. Since EHR data limits insight into individual treatment decisions, we used a simplified approach for the minimum prevention: for patients requiring a lipid lowering agent because they were not meeting their LDL goal, or those with a history of CAD, ischemic stroke, or diabetes, appropriate treatment change was defined as either (i) initiating statin prescription if no statin was prescribed earlier, (ii) increasing current statin prescription dose [different statin type dosages were harmonized based on comparison factors from trials evaluating statin efficacy (22)], or (iii) initiating a LDL-lowering drug from a different class such as ezetimibe, fibrate, or a PCSK9-inhibitor; all other combinations were considered as inappropriate treatment. For patients meeting their LDL goal, every situation of medication before/after measurement was considered as appropriate treatment.

Because of the individualized screening and treatment recommendations depending on patients’ age, comorbidities, life expectancy, and functional status (15, 23), we used a simplified approach to define goals for screening/evaluation and treatment of diabetes. We defined a HbA1c goal of 7% for patients <65 years, 7.5% for patients ≥ 65 years before ICH, and 8% for patients ≥65 years after ICH. Furthermore, we defined the treatment change following HbA1c measurement as appropriate, inappropriate or unknown. If HbA1c exceeded the target, intensification of therapy ascertained by chart review was considered appropriate treatment. For patients without prior insulin prescription, addition or dose increase of an oral antidiabetic drug or initiation of insulin therapy was defined as treatment intensification. For patients with prior insulin treatment, we could not capture appropriate treatment change because intensification of the insulin administration plan is not reliably reflected in the EHR, therefore these situations were defined as unknown. All other situations were defined as inappropriate treatment changes.

### Social determinants of health

We explored the association between socioeconomic disadvantage and health care utilization before and after ICH. As a proxy for individual disadvantage, we used the previously validated United States nation-wide area deprivation index (ADI) (24–26), which is composed of 17 education, employment, housing quality, and poverty measures, divided into deciles with higher values indicating greater deprivation. We gathered the neighborhood-level ADI of the census block group of each individual’s address at the time of ICH from the Neighborhood Atlas.^1^

### Statistical methods and software used

Measures are reported as mean and standard deviation (SD) if normally distributed, otherwise as median and interquartile range (IQR). For comparison of categorical variables, Chi-square test and Fisher’s exact test (in case of cell counts <5) were used; for comparison of continuous variables, unpaired *t*-test or Wilcoxon signed-rank test (for normally and non-normally distributed variables, repectively) were used. The association between ADI and management before, during and after hospitalization was assessed with logistic regression models adjusted for age, sex, race, and in models that evaluated LDL and HbA1c measurements for presence of coronary artery disease and diabetes, respectively. All analyses were performed in RStudio 2022.07.0 with R version 4.2.1 on Mac OS X (aarch64-apple-darwin20) (27).

## Results

**Figure 1:**
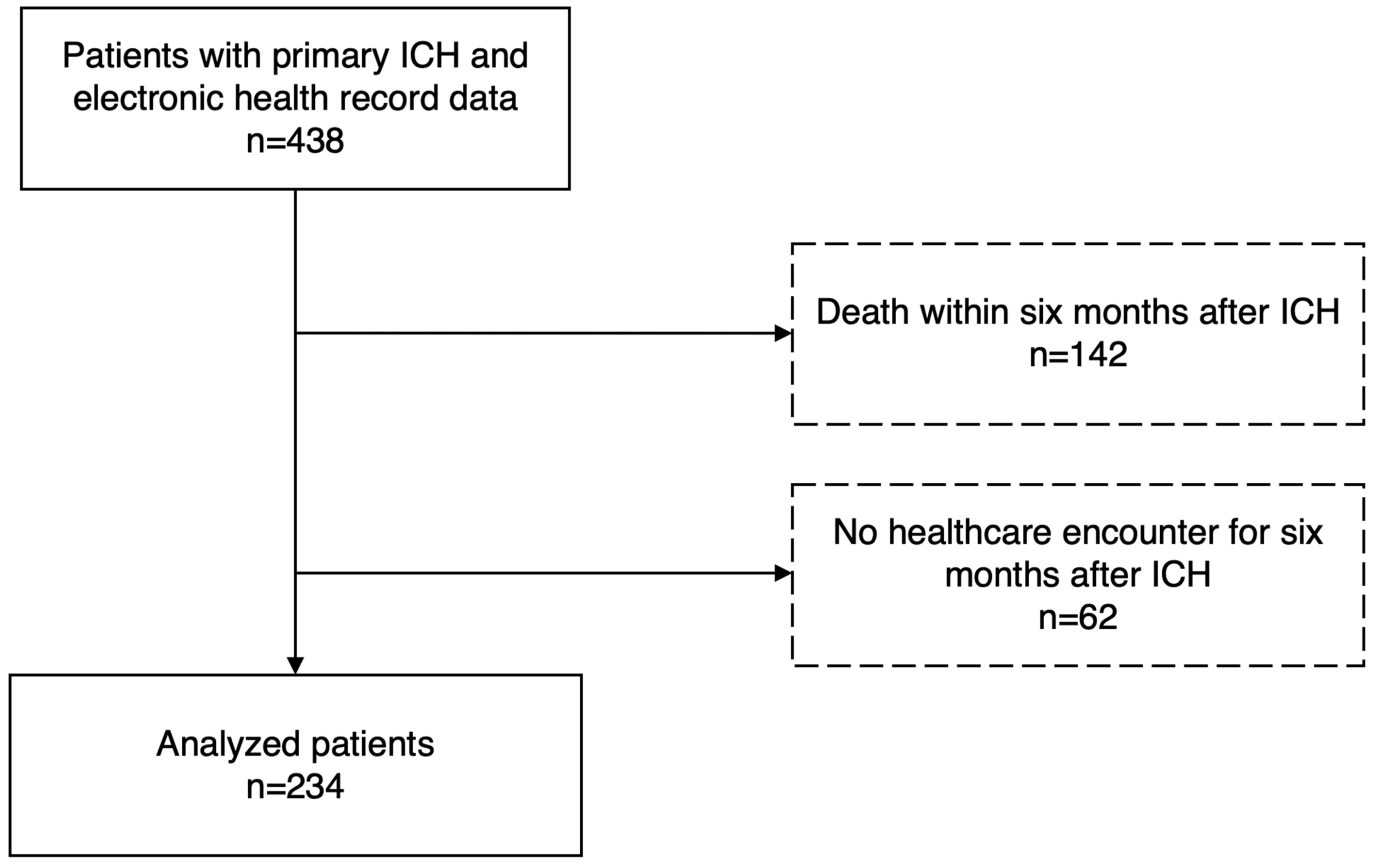
Study flow. From the prospective longitudinal cohort of patients with intracerebral hemorrhage and available electronic health care data at Massachusetts General Hospital those who survived more than 6 months and received care within the Massachusetts General Hospital provider network were included.

Out of 438 patients from the MGH ICH study with available electronic health record data, 234 patients [median (IQR) 71 (61–79) years, 42% female] remained for analysis after exclusion of those who did not survive or did not utilize EHR-accessible healthcare within 6 months following ICH (Figure 1 and Table 1). ADI was available for 220 patients with a median nation-wide ADI rank decile of 1.8 (IQR 0.9–2.7).

**Table 1:** Baseline characteristics of the 234 analyzed patients with intracerebral hemorrhage.

	All (*n* = 234)	Low ADI (*n* = 114)	High ADI (*n* = 106)	*p*
Age, years, median (IQR)	71 (61–79)	72 (64–80)	69 (59–79)	0.12
Female, *n* (%)	99 (42.3)	53 (46.5)	44 (41.5)	0.46
ADI decile, median (IQR)	1.8 (0.9–2.7)	1.0 (0.5–1.5)	2.7 (2.3–3.5)	<0.001
Race				
White, *n* (%)	192 (82.1)	100 (87.7)	81 (76.4)	0.02
Asian, *n* (%)	16 (6.8)	7 (6.1)	8 (7.5)	0.67
Black, *n* (%)	14 (6.0)	4 (3.5)	8 (7.5)	0.24
Native American, *n* (%)	1 (0.4)	0 (0)	1 (0.9)	0.48
Other, *n* (%)	8 (3.4)	3 (2.6)	5 (4.7)	0.49
Unknown, *n* (%)	3 (1.3)	0 (0)	3 (2.8)	0.11
Ethnicity				
Hispanic, *n* (%)	16 (6.8)	4 (3.5)	11 (10.4)	0.04
Not Hispanic, *n* (%)	207 (88.5)	104 (91.2)	91 (85.8)	0.21
Unknown, *n* (%)	11 (4.7)	6 (5.3)	4 (3.8)	0.60
Hemorrhage location				
Deep, *n* (%)	118 (50.4)	49 (43.0)	62 (58.5)	0.02
Lobar, *n* (%)	105 (44.9)	59 (51.8)	40 (37.7)	0.04
Cerebellar, *n* (%)	10 (4.3)	5 (4.4)	4 (3.8)	0.99
Mixed, *n* (%)	1 (0.4)	1 (0.9)	0 (0)	0.99
Cerebrovascular risk factors				
Hypertension, *n* (%)	200 (86.2)	97 (85.8)	92 (87.6)	0.70
Hypercholesterolemia, *n* (%)	123 (53.0)	66 (57.9)	52 (49.5)	0.21
Diabetes, *n* (%)	54 (23.4)	23 (20.4)	30 (28.6)	0.16
Atrial fibrillation, *n* (%)	47 (20.3)	28 (24.6)	18 (17.3)	0.19
Coronary artery disease, *n* (%)	33 (14.3)	14 (12.3)	16 (15.4)	0.51
Ischemic CVA, *n* (%)	31 (13.4)	14 (12.4)	16 (15.4)	0.52

Patients were divided into low and high deprivation by median area deprivation index (ADI).

### Blood pressure

#### Evaluation patterns before, during, and after hospitalization

**Figure 2:**
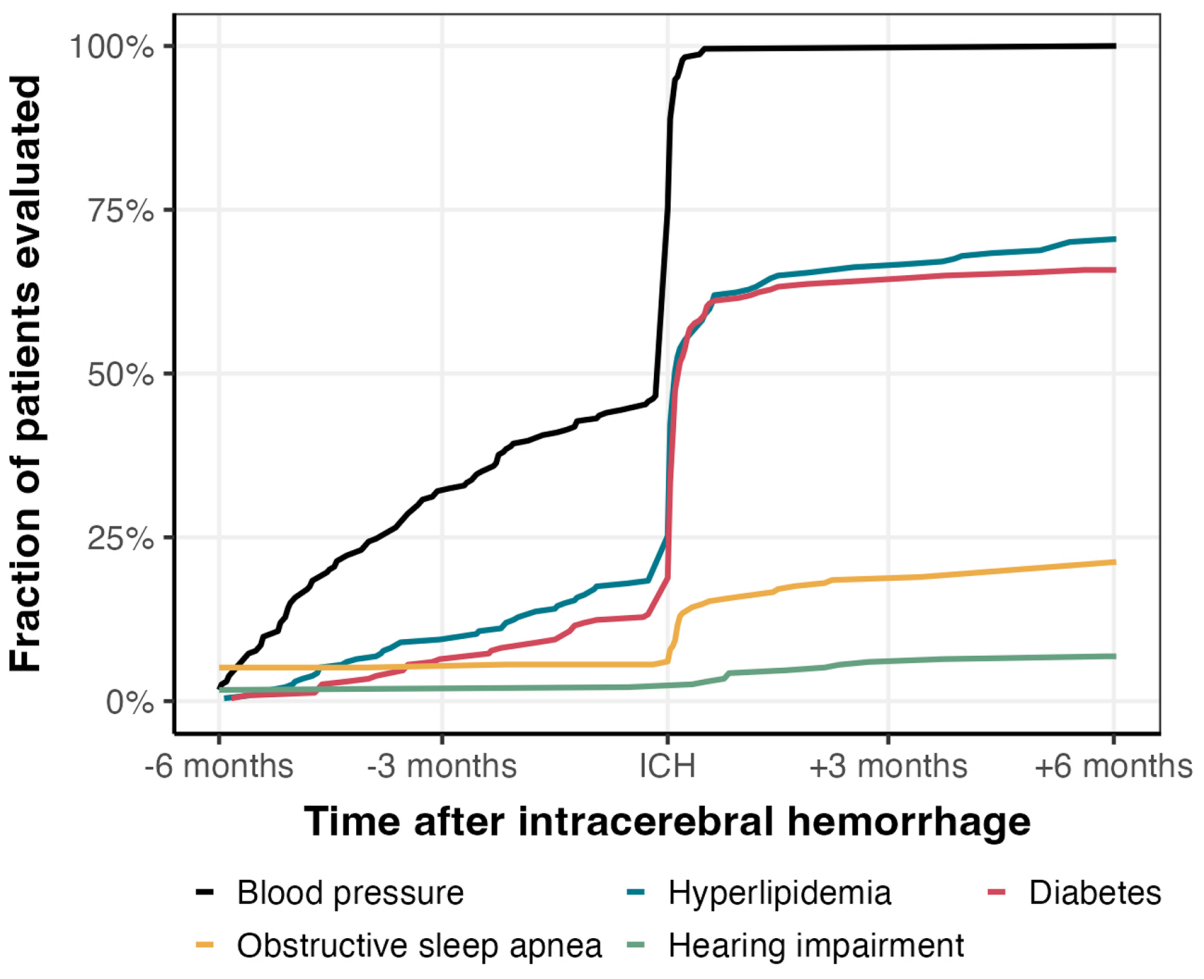
Comorbidity management in the 12 months surrounding ICH. Proportion of patients that were evaluated for hypertension, hyperlipidemia, diabetes, obstructive sleep apnea, and hearing impairment.

In total, 109 patients (47%) had one or more recorded BP measurement before ICH, whereas 214 (91%) had a BP measurement after ICH (Figure 2). Of the patients that had at least one class of BP lowering medication prescribed before ICH (n = 108, 46%), only 75 had one or more recorded BP measurements in the 6 months before ICH.

Among the 109 patients with BP readings before ICH, 51 patients were started on a new antihypertensive drug before ICH. When comparing the number of antihypertensives before and after ICH hospitalization, we found a mean increase of 1.3 antihypertensives with 153 (65%) patients having at least one more, 65 patients having the same number, and only 16 patients having fewer antihypertensives prescribed after ICH than before.

#### Association between social determinants of health and management

Higher ADI was associated with a lower probability of BP measurements before hospitalization, independent of presence of coronary artery disease and diabetes [OR 0.94, 95% CI (0.90, 0.99) per one ADI decile]. We found no association between the ADI and antihypertensives before and after hospitalization or with the difference in antihypertensives before and after hospitalization (all *p* > 0.2).

### Hyperlipidemia and diabetes

#### Evaluation patterns before, during, and after hospitalization

**Figure 3:**
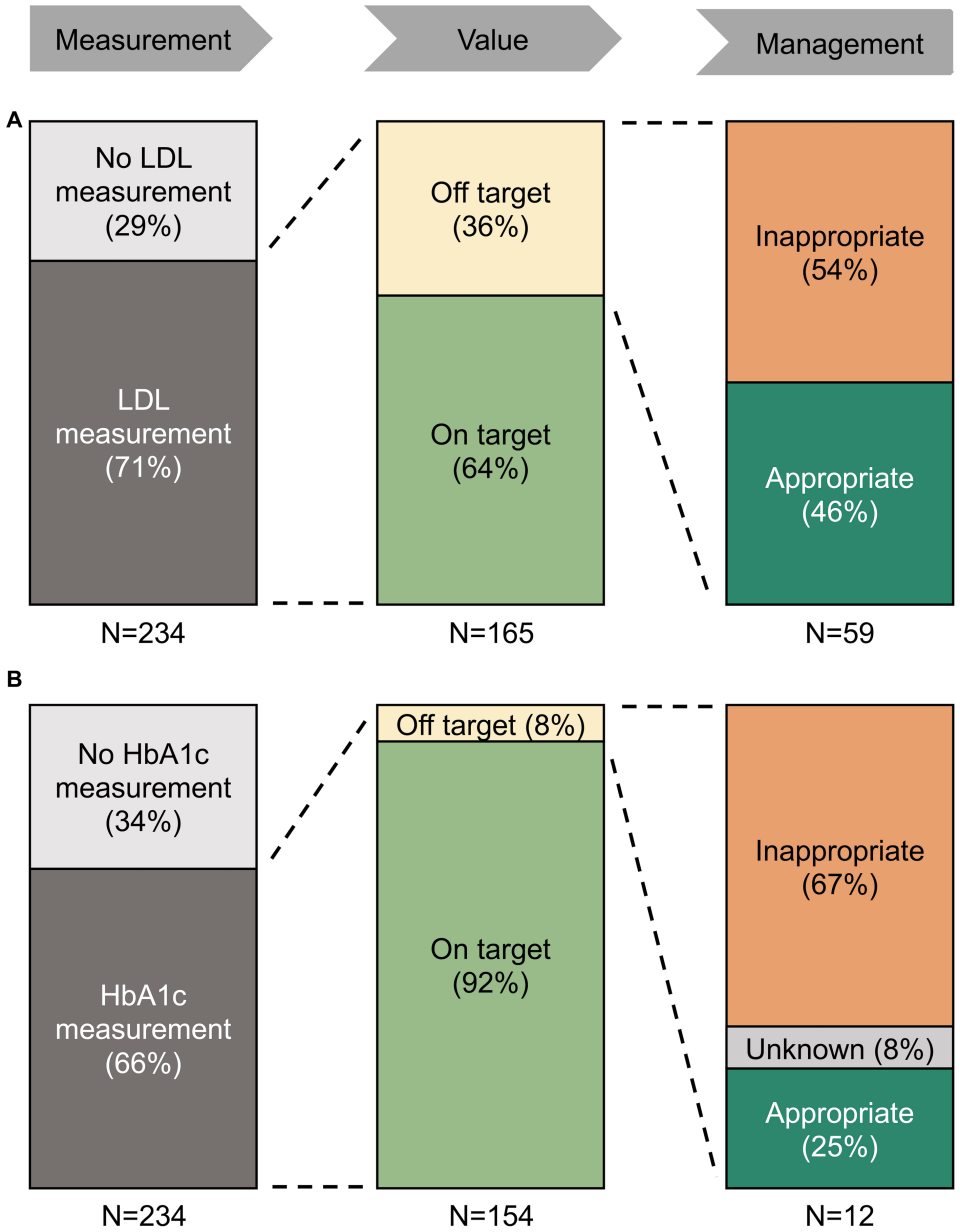
Treatment patterns of hyperlipidemia **(A)** and diabetes **(B)** in the 12 months surrounding ICH. Proportion of ICH patients with LDL measurements, target values, and treatment changes. Among patients with off-target values of LDL and HbA1c, the majority did not receive appropriate management. There was no association between area deprivation index and the achievement of LDL goals or the change in treatment.

136 (58%) and 137 (59%) of the patients had LDL and HbA1c measurements during hospitalization or within 6 months before admission, respectively, with a sharp increase during hospitalization (Figure 2). Of the remaining patients without measurements within 6 months before admission and discharge, only 29 (12%) and 17 (7%) of the patients received LDL and HbA1c measurements within 6 months after hospitalization, respectively (Figure 2). Altogether, we found one or more LDL or HbA1c measurement within the 12 months surrounding ICH in 165 (71%) and 154 (66%) of patients, respectively (Figure 3).

#### Management strategies

Of the patients with LDL measurements, 106 (64%) were on their LDL target at least once in the 12 months surrounding ICH. Of the remaining 59 patients with only off-target LDL measurements, we found an appropriate change in treatment in 27 (46%) of the patients (Figure 3). Of the patients with HbA1c measurements, 142 (85%) met their HbA1c target at least once in the 12 months surrounding ICH. Of the remaining 12 patients with only off-target HbA1c, we found an appropriate change in treatment in three (25%) patients (Figure 3).

#### Association between social determinants of health and management

There was no association between ADI and LDL and HbA1c measurement in the 12 months surrounding ICH overall (*p* > 0.2 for all). However, higher ADI was associated with a lower probability of LDL and HbA1c measurements before hospitalization, independent of presence of coronary artery disease or diabetes, respectively [OR 0.95, 95% CI (0.91, 0.98) and 0.96, 95% CI (0.93, 0.99) per one ADI decile]. We found no association between ADI and achievement of LDL goals or treatment patterns.

### Obstructive sleep apnea and hearing impairment

#### Evaluation patterns during and after hospitalization

We found evidence of OSA evaluation within 6 months in 47 (23%) of the 207 patients without documented OSA prior to ICH, of which 18 had a referral/appointment for a sleep study within the 6 months following ICH and 29 had a new mention of OSA in their notes or EHR problem list (Figure 2). We found evidence of evaluation for hearing impairment in 22 (9%) patients of the 212 patients without documentation of hearing impairment prior to ICH: 9 had a referral or appointment for audiology and 7 had a new documentation of hearing aids within the 6 months following ICH (Figure 2).

#### Association between social determinants of health and management

Higher ADI was associated with decreased probability of OSA diagnosis prior to hospitalization [OR 0.96 95% CI (0.93, 0.99) per one decile nation-wide rank], but not with OSA evaluation after ICH (*p* > 0.2). There was no association between the ADI and hearing impairment before ICH or evaluation for hearing impairment after ICH (both *p* > 0.2).

## Discussion

We ascertained current provider practices regarding evaluation and management of hyperlipidemia, diabetes, obstructive sleep apnea, and hearing impairment in 234 ICH survivors from a large academic institution and the associations between evaluation rates and social determinants of health. We found infrequent measurements of BP, LDL and HbA1c before hospitalization, associated with socioeconomic disadvantage. Even beyond health disparities, LDL and HbA1c were measured in just half of eligible ICH patients during hospitalization and increased only slightly after hospitalization. Evaluating OSA and hearing impairment, comorbidities that are highly prevalent in ICH patients (1, 12, 28), roughly 10% of patients carried a prior diagnosis and only an additional 23 and 8%, respectively, were evaluated following ICH hospitalization.

Individuals with higher ADI had a lower chance of BP, LDL, and HbA1c measurements and OSA diagnosis before hospitalization, but not during or after hospitalization. These results suggest that health care disparities are influenced by social determinants of health before ICH, but also that the acute ICH hospitalization offers an opportunity to correct health care disparities among ICH survivors, even though a standardized approach for management of cerebrovascular and adverse brain health risk factors perhaps not directly related to ICH was lacking in our cohort. Future interventions during ICH hospitalization for evaluation and management of common risk factors and comorbidities to mitigate health care disparities and potentially change brain health outcomes may be an effective strategy.

Our analysis focused on comorbidities that are highly prevalent in ICH patients (6, 10, 29) and whose management is backed by solid evidence and established routine in the general population (9, 30). Our findings may also apply to other comorbidities that contribute to long-term outcomes. Because of the high mortality and severe disability in patients with ICH, health care providers might be tempted to focus on only the most pressing matters during acute hospitalization. But deferral of risk factor evaluation might mean dismissal: most of the assessments after ICH happened during hospitalization, and patients were unlikely to be evaluated as far out as 6 months post-ICH. Hyperlipidemia and diabetes strongly contribute to the excess risk for major cardiovascular and cerebrovascular events in ICH survivors (10, 12), and elevated values have well-established consequences. Recognition of OSA is particularly important because it increases BP, the most important risk factor for recurrent ICH (12). The risk of OSA can be easily assessed with clinical risk scores, such as the STOP-BANG score (31), or even with risk factors extracted from the EHR (32), and a subsequent sleep study bears low risk (33). Hearing impairment, highly prevalent among stroke patients (13), influences recovery and rehabilitation after ICH (34) and can be screened with established questionnaires or a handheld hearing screener (35).

Except for BP measurement and intervention, there is currently no systematic approach for screening these risk factors in ICH patients (9), partly because there is not evidence that intervening improves secondary prevention in ICH patients specifically. The low absolute incidence of ICH limits traditional trials in the absence of large and costly multi-center efforts, but alternative strategies, such as EHR-based intervention studies, could improve screening rates and facilitate outcome tracking. Such interventions could be designed to modify provider behavior through EHR-based prompts that show additional information, such as last measurements, known diagnoses, or automatic risk score calculation, and have been shown to be effective in other settings (36). Effects are measured by either by randomizing patients (37) or providers (38), or by comparing outcomes before and after intervention (39). Since EHR systems are highly prevalent in the US, it may be feasible to explore whether a recommendation to screen risk factors and comorbidities in ICH patients improves outcomes.

Our study has limitations. First, EHR data limits insight into individual patient’s medical history, particularly parts that are outside the MGH provider network. There might have been individual reasons for lack of evaluation or management characterized as inappropriate that our analyses could not capture. Second, subsequent measurements and referrals could have been made at providers outside the MGH network, thus we might have underestimated their rates. We have tried to mitigate that bias by restricting our cohort to patients that had at least one encounter with the system after 6 months or later. Third, we used neighborhood-level ADI as a proxy for individual access to healthcare which is a crude generalization, especially about information about patients’ own education level, one of the main determinants of health and access to health care (40); however, the ADI has been validated in previous studies as a reliable proxy for SDOH (25). Furthermore, we might have missed an association between ADI and health care utilization after ICH due to the low absolute number of LDL and HbA1c measurements after hospitalization. There are no obvious reasons why health care disparities should cease after ICH hospitalization, but it is possible that the relatively resource-rich environment surrounding post-ICH rehabilitation at least partially mitigates such disparities. The low ADI in our cohort compared to the rest of the US (1.8 out of 10) suggests that many patients in our cohort had good access to health care and other resources and thus limits the generalizability of our single-center study, however disparities might be even magnified in more deprived environments. Fourth, our study does not incorporate a control group, which could demonstrate whether the identified screening deficiencies are specific to ICH patients, or extend to a broader patient population. However, our primary objective was to delineate areas of potential improvement specifically in ICH patient care, within the framework of existing American Heart Association guidelines specific to post-ICH care. Moreover, the selection of an appropriate control group, characterized by comparable demographics, published guidelines, and equally severe disease conditions not directly linked to hyperlipidemia or diabetes, presents a significant challenge. Finally, we do not directly know whether better management of the proposed risk factors and comorbidities will improve long-term outcomes in ICH survivors, a population with a lower life expectancy and more functional impairment than the general population or even ischemic stroke survivors.

In conclusion, our EHR data indicates that there is room for improvement for the management of hyperlipidemia, diabetes, OSA, and hearing impairment in ICH survivors and that the ICH hospitalization is a prime opportunity to mitigate care biases influenced by social determinants of health. Future studies should assess whether systematic comorbidity management improves long-term outcomes in this highly vulnerable collective.

## Data availability statement

The datasets presented in this article are not readily available because of ethical and privacy restrictions. Requests to access the datasets should be directed to the corresponding author.

## Ethics statement

The studies involving human participants were reviewed and approved by the Mass General Brigham IRB (#2021P001597). The patients/participants provided their written informed consent to participate in this study.

## Author contributions

EM, LP, and ASG gathered the data and performed the data analysis. AB and JR performed the enrollment of patients in this cohort. EM and NZ wrote the manuscript with input from all co-authors. NY contributed to the final version of the manuscript. CA supervised the data extraction, analysis, and writing. All authors discussed the results and commented on the manuscript.

## Funding

CA is supported by the NIH R01NS103924, U01NS069673, AHA 18SFRN34250007, AHA-Bugher 21SFRN812095, and the MGH McCance Center for Brain Health for this work. JR receives research grants from the National Institutes of Health and the American Heart Association-Bugher Foundation. NY receives funding from the MGH Heitman Young Investigator Award and the American Heart Association-Bugher Foundation.

## Conflict of interest

CA has received sponsored research support from Bayer AG and has consulted for ApoPharma. JR has consulted for Boehringer Ingelheim and Takeda.

The remaining authors declare that the research was conducted in the absence of any commercial or financial relationships that could be construed as a potential conflict of interest.

## Publisher’s note

All claims expressed in this article are solely those of the authors and do not necessarily represent those of their affiliated organizations, or those of the publisher, the editors and the reviewers. Any product that may be evaluated in this article, or claim that may be made by its manufacturer, is not guaranteed or endorsed by the publisher.

## Footnotes

^1^ https://www.neighborhoodatlas.medicine.wisc.edu

## References

[1] Kittner SJ Sekar P Comeau ME Anderson CD Parikh GY Tavarez T. Ethnic and racial variation in intracerebral hemorrhage risk factors and risk factor burden. JAMA Netw Open. (2021) 4:e2121921. doi: 10.1001/jamanetworkopen.2021.21921, PMID: 34424302 PMC8383133

[2] Abramson JR Castello JP Keins S Kourkoulis C Rodriguez-Torres A Myserlis EP. Biological and social determinants of hypertension severity before Vs after intracerebral hemorrhage. Neurology. (2022) 98:e1349–60. doi: 10.1212/wnl.0000000000200003, PMID: 35131909 PMC8967426

[3] Castello JP Pasi M Abramson JR Rodriguez-Torres A Marini S Demel S. Contribution of racial and ethnic differences in cerebral small vessel disease subtype and burden to risk of cerebral hemorrhage recurrence. Neurology. (2021) 96:e2469–80. doi: 10.1212/wnl.0000000000011932, PMID: 33883240 PMC8205476

[4] Leasure AC King ZA Torres-Lopez V Murthy SB Kamel H Shoamanesh A. Racial/ethnic disparities in the risk of intracerebral hemorrhage recurrence. Neurology. (2020) 94:e314–22. doi: 10.1212/wnl.0000000000008737, PMID: 31831597 PMC7108806

[5] Walsh KB Woo D Sekar P Osborne J Moomaw CJ Langefeld CD. Untreated hypertension: a powerful risk factor for lobar and nonlobar Intracerebral hemorrhage in whites, blacks, and Hispanics. Circulation. (2016) 134:1444–52. doi: 10.1161/circulationaha.116.024073, PMID: 27737957 PMC5123682

[6] Schneider BC Gross AL Bangen KJ Skinner JC Benitez A Glymour MM. Association of vascular risk factors with cognition in a multiethnic sample. J Gerontol B Psychol Sci Soc Sci. (2015) 70:532–44. doi: 10.1093/geronb/gbu040, PMID: 24821298 PMC4462669

[7] Sorrel JE Bishop CE Spankovich C Su D Valle K Seals S. Relationship of stroke risk and hearing loss in African Americans: the Jackson heart study. Laryngoscope. (2018) 128:1438–44. doi: 10.1002/lary.26896, PMID: 28990660 PMC5891391

[8] Biffi A Anderson CD Battey TW Ayres AM Greenberg SM Viswanathan A. Association between blood pressure control and risk of recurrent intracerebral hemorrhage. JAMA. (2015) 314:904–12. doi: 10.1001/jama.2015.10082, PMID: 26325559 PMC4737594

[9] Greenberg SM Ziai WC Cordonnier C Dowlatshahi D Francis B Goldstein JN. 2022 guideline for the management of patients with spontaneous intracerebral hemorrhage: a guideline from the American Heart Association/American Stroke Association. Stroke. (2022) 53:e282–361. doi: 10.1161/str.0000000000000407, PMID: 35579034

[10] Castello JP Teo KC Abramson JR Keins S Takahashi CE Leung IYH. Long-term blood pressure variability and major adverse cardiovascular and cerebrovascular events after Intracerebral hemorrhage. J Am Heart Assoc. (2022) 11:e024158. doi: 10.1161/jaha.121.024158, PMID: 35253479 PMC9075304

[11] Biffi A Bailey D Anderson CD Ayres AM Gurol EM Greenberg SM. Risk factors associated with early Vs delayed dementia after intracerebral hemorrhage. JAMA Neurol. (2016) 73:969–76. doi: 10.1001/jamaneurol.2016.0955, PMID: 27295605 PMC5327781

[12] Geer JH Falcone GJ Vanent KN Leasure AC Woo D Molano JR. Obstructive sleep apnea as a risk factor for intracerebral hemorrhage. Stroke. (2021) 52:1835–8. doi: 10.1161/strokeaha.120.033342, PMID: 33827242 PMC8085039

[13] O’halloran R Worrall LE Hickson L. The number of patients with communication related impairments in acute hospital stroke units. Int J Speech Lang Pathol. (2009) 11:438–49. doi: 10.3109/17549500902741363, PMID: 21271921

[14] Abramson JR Castello JP Keins S Kourkoulis C Gurol ME Greenberg SM. Association of symptomatic hearing loss with functional and cognitive recovery 1 year after Intracerebral hemorrhage. J Stroke. (2022) 24:303–6. doi: 10.5853/jos.2022.00836, PMID: 35677987 PMC9194545

[15] American Diabetes Association Professional Practice Committee. 6. Glycemic targets: standards of medical care in Diabetes-2022. Diabetes Care. (2022) 45:S83–96. doi: 10.2337/dc22-S006 34964868

[16] Grundy SM Stone NJ Bailey AL Beam C Birtcher KK Blumenthal RS. 2018 Aha/Acc/Aacvpr/Aapa/Abc/Acpm/Ada/Ags/Apha/Aspc/Nla/Pcna guideline on the management of blood cholesterol: executive summary: a report of the American college of cardiology/American Heart Association task force on clinical practice guidelines. J Am Coll Cardiol. (2019) 73:3168–209. doi: 10.1016/j.jacc.2018.11.002, PMID: 30423391

[17] Aaronson JA Van Bennekom CA Hofman WF Van Bezeij T Van Den Aardweg JG Groet E. Obstructive sleep apnea is related to impaired cognitive and functional status after stroke. Sleep. (2015) 38:1431–7. doi: 10.5665/sleep.4984, PMID: 25669178 PMC4531411

[18] Hale E Gottlieb E Usseglio J Shechter A. Post-stroke sleep disturbance and recurrent cardiovascular and cerebrovascular events: a systematic review and Meta-analysis. Sleep Med. (2023) 104:29–41. doi: 10.1016/j.sleep.2023.02.019, PMID: 36889030 PMC10098455

[19] Boulos MI Dharmakulaseelan L Brown DL Swartz RH. Trials in sleep apnea and stroke: learning from the past to direct future approaches. Stroke. (2021) 52:366–72. doi: 10.1161/strokeaha.120.031709 33349009

[20] Edwards DF Hahn MG Baum CM Perlmutter MS Sheedy C Dromerick AW. Screening patients with stroke for rehabilitation needs: validation of the post-stroke rehabilitation guidelines. Neurorehabil Neural Repair. (2006) 20:42–8. doi: 10.1177/1545968305283038, PMID: 16467277

[21] Skolarus LE Lisabeth LD Morgenstern LB Burgin W Brown DL. Sleep apnea risk among Mexican American and non-Hispanic white stroke survivors. Stroke. (2012) 43:1143–5. doi: 10.1161/strokeaha.111.638387, PMID: 22156693 PMC3314716

[22] Weng TC Yang YH Lin SJ Tai SH. A systematic review and Meta-analysis on the therapeutic equivalence of statins. J Clin Pharm Ther. (2010) 35:139–51. doi: 10.1111/j.1365-2710.2009.01085.x, PMID: 20456733

[23] American Diabetes Association Professional Practice Committee. 13. Older adults: standards of medical care in Diabetes-2022. Diabetes Care. (2022) 45:S195–207. doi: 10.2337/dc22-S013, PMID: 34964847 PMC8935395

[24] Hu J Kind AJH Nerenz D. Area deprivation index predicts readmission risk at an urban teaching hospital. Am J Med Qual. (2018) 33:493–501. doi: 10.1177/1062860617753063, PMID: 29357679 PMC6027592

[25] Kind AJ Jencks S Brock J Yu M Bartels C Ehlenbach W. Neighborhood socioeconomic disadvantage and 30-day rehospitalization: a retrospective cohort study. Ann Intern Med. (2014) 161:765–74. doi: 10.7326/m13-2946, PMID: 25437404 PMC4251560

[26] Kind AJH Buckingham WR. Making neighborhood-disadvantage metrics accessible – the neighborhood atlas. N Engl J Med. (2018) 378:2456–8. doi: 10.1056/nejmp1802313, PMID: 29949490 PMC6051533

[27] Rstudio Team. Rstudio: integrated development environment for R. Boston, MA: Rstudio, PBC (2021).

[28] Garvey JF Pengo MF Drakatos P Kent BD. Epidemiological aspects of obstructive sleep apnea. J Thorac Dis. (2015) 7:920–9. doi: 10.3978/j.issn.2072-1439.2015.04.52, PMID: 26101650 PMC4454867

[29] Poon MT Fonville AF Al-Shahi Salman R. Long-term prognosis after intracerebral Haemorrhage: systematic review and meta-analysis. J Neurol Neurosurg Psychiatry. (2014) 85:660–7. doi: 10.1136/jnnp-2013-306476, PMID: 24262916

[30] Arnett DK Blumenthal RS Albert MA Buroker AB Goldberger ZD Hahn EJ. 2019 ACC/AHA guideline on the primary prevention of cardiovascular disease: executive summary: a report of the American college of cardiology/American heart association task force on clinical practice guidelines. Circulation. (2019) 140:e563–95. doi: 10.1161/cir.0000000000000677, PMID: 30879339 PMC8351755

[31] Chung F Abdullah HR Liao P. Stop-Bang questionnaire: a practical approach to screen for obstructive sleep apnea. Chest. (2016) 149:631–8. doi: 10.1378/chest.15-0903 26378880

[32] Ustun B Westover MB Rudin C Bianchi MT. Clinical prediction models for sleep apnea: the importance of medical history over symptoms. J Clin Sleep Med. (2016) 12:161–8. doi: 10.5664/jcsm.5476, PMID: 26350602 PMC4751423

[33] Blattner M Dunham K Thomas R Ahn A. A protocol for mitigating safety events in a sleep laboratory. J Clin Sleep Med. (2021) 17:1355–61. doi: 10.5664/jcsm.9190, PMID: 33660613 PMC8314629

[34] Landi F Onder G Cesari M Zamboni V Russo A Barillaro C. Functional decline in frail community-dwelling stroke patients. Eur J Neurol. (2006) 13:17–23. doi: 10.1111/j.1468-1331.2006.01116.x, PMID: 16420389

[35] Koohi N Vickers DA Utoomprurkporn N Werring DJ Bamiou DE. A hearing screening protocol for stroke patients: an exploratory study. Front Neurol. (2019) 10:842. doi: 10.3389/fneur.2019.00842, PMID: 31447763 PMC6691813

[36] Bejjanki H Mramba LK Beal SG Radhakrishnan N Bishnoi R Shah C. The role of a best practice alert in the electronic medical record in reducing repetitive lab tests. Clinicoecon Outcomes Res. (2018) 10:611–8. doi: 10.2147/ceor.s167499, PMID: 30323637 PMC6181108

[37] Wilson FP Martin M Yamamoto Y Partridge C Moreira E Arora T. Electronic health record alerts for acute kidney injury: multicenter, randomized clinical trial. BMJ. (2021) 372:m4786. doi: 10.1136/bmj.m4786 33461986 PMC8034420

[38] Juszczyk D Charlton J Mcdermott L Soames J Sultana K Ashworth M. Electronically delivered, multicomponent intervention to reduce unnecessary antibiotic prescribing for respiratory infections in primary care: a cluster randomised trial using electronic health records-reduce trial study original protocol. BMJ Open. (2016) 6:e010892. doi: 10.1136/bmjopen-2015-010892, PMID: 27491663 PMC4985802

[39] Laxmisan A Sittig DF Pietz K Espadas D Krishnan B Singh H. Effectiveness of an electronic health record-based intervention to improve follow-up of abnormal pathology results: a retrospective record analysis. Med Care. (2012) 50:898–904. doi: 10.1097/MLR.0b013e31825f6619, PMID: 22929995 PMC3444625

[40] David M. C. Adriana L.-M. (2006). Education and health: evaluating theories and evidence.

