# Supplementary material for: Disparities in brain health comorbidity management in intracerebral hemorrhage
Source: Front Neurol. 2023 Jun 8;14:1194810. doi: 10.3389/fneur.2023.1194810 (PMC10285101; doi:10.3389/fneur.2023.1194810)
Supplement: Supplementary file 1 [file Table_1.DOCX]

## Supplemental Methods

## LDL and HbA1c measurements

We were interested in the rate of LDL and HbA1c measurements and appropriate management of hyperlipidemia and diabetes during hospitalization and in the six months following ICH, taking into account measurements and treatment changes in the six months before ICH.

According to current guidelines, adults over the age of 40 years should be *routinely* assessed for cardiovascular risk factors and for adults between 20 to 40 years it is *reasonabl*e to assess cardiovascular risk factors every 4 to 6 years.^1^ Thus, given the age of the ICH population, it is reasonable to assume that LDL should be measured at least once during the 12 months surrounding ICH. In individuals without diabetes, screening for diabetes should be performed at least every three years beginning at the age of 35 years and can be screened for more frequently in individuals with a higher risk status.^2^ In individuals with diabetes, HbA1c measurement is recommended twice a year if they are meeting their glycemic targets and every three months if not.^3^ Similarly to LDL, we assumed that because of the overall high cardiovascular risk, HbA1c should be measured at least once during the 12 months surrounding ICH.

According to current guidelines, HbA1c goal is dependent on age and functional status:^3, 4^ for older adults (≥65 years) who “*are otherwise healthy with few coexisting chronic illnesses and intact cognitive function and functional status”,* the target HbA1c is < 7-7.5%, while for those with *“multiple coexisting chronic illnesses, cognitive impairment, or functional dependence”* the target HbA1c is 8%;^4^ for patients <65 years, HbA1c goal < 7% is appropriate.^3^

## Obstructive sleep apnea and hearing impairment

We sought to ascertain the ratio of patients getting referred or scheduled for a sleep study for evaluation of obstructive sleep apnea of those without evidence of OSA. To identify patients with prior diagnosis of obstructive sleep apnea, we searched for “apnea” in the diagnosis list and for “apnea”, “cpap”, or “osa” in problems and for “osa” and “sleep apnea” in notes prior to index event. We further aimed to assess the ratio of patients getting an audiology referral after ICH of those who could potentially benefit from hearing aids. Considering the age and comorbidities of ICH patients, we assumed that all patients could benefit from audiology except individuals with audiology referrals/appointments and those with hearing aids prior to index event. To identify patients using hearing aids, we searched for the term “hearing aid” in notes 7 days prior to index event (to account for documentation of hearing aids during acute hospitalization).

## Software used

We used Structured Query Language (SQL) to gather patients’ encounters, diagnosis lists, problems, notes, appointments, referrals, procedures, and medication prescriptions from the MGH electronic data warehouse. Whole word regular expressions were used to search for patterns followed by manual curation of the extracted text. For data extraction, curation, preparation, statistical analysis, and figure generation, we used RStudio 2022.07.0 with R version 4.2.1 on Mac OS X (aarch64-apple-darwin20).

## Supplemental References

1. Arnett DK, Blumenthal RS, Albert MA, et al. 2019 ACC/AHA Guideline on the Primary Prevention of Cardiovascular Disease: Executive Summary: A Report of the American College of Cardiology/American Heart Association Task Force on Clinical Practice Guidelines. *Circulation* 2019; 140: e563-e595. 20190317. DOI: 10.1161/CIR.0000000000000677.

2. American Diabetes Association Professional Practice C, American Diabetes Association Professional Practice C, Draznin B, et al. 2. Classification and Diagnosis of Diabetes: Standards of Medical Care in Diabetes-2022. *Diabetes Care* 2022; 45: S17-S38. DOI: 10.2337/dc22-S002.

3. American Diabetes Association Professional Practice C, American Diabetes Association Professional Practice C, Draznin B, et al. 6. Glycemic Targets: Standards of Medical Care in Diabetes-2022. *Diabetes Care* 2022; 45: S83-S96. 2021/12/30. DOI: 10.2337/dc22-S006.

4. American Diabetes Association Professional Practice C, American Diabetes Association Professional Practice C, Draznin B, et al. 13. Older Adults: Standards of Medical Care in Diabetes-2022. *Diabetes Care* 2022; 45: S195-S207. 2021/12/30. DOI: 10.2337/dc22-S013.
